# Cancer Treatment During Pregnancy and Maternal and Infant Outcomes: A Descriptive Cohort Study Using a Japanese Claims Database

**DOI:** 10.1002/pds.70508

**Published:** 2026-10-16

**Authors:** Masami Tsuchiya, Tomofumi Ishikawa, Takamasa Sakai, Satoko Hori, Taku Obara

**Affiliations:** ^1^ Division of Drug Informatics Keio University Faculty of Pharmacy Tokyo Japan; ^2^ Laboratory of Biomolecule and Pathophysiological Chemistry, Graduate School of Pharmaceutical Sciences Tohoku University Sendai Miyagi Japan; ^3^ Drug Informatics, Faculty of Pharmacy Meijo University Nagoya Aichi Japan; ^4^ Department of Pharmaceutical Sciences Tohoku University Hospital Sendai Miyagi Japan; ^5^ Division of Preventive Medicine and Epidemiology Tohoku University Tohoku Medical Megabank Organization Sendai Miyagi Japan; ^6^ Department of Clinical Epidemiology, Graduate School of Medicine Tohoku University Sendai Miyagi Japan

**Keywords:** administrative data, antineoplastic agents, cancer during pregnancy, claims, congenital abnormalities, pharmacoepidemiology, pregnancy

## Abstract

**Purpose:**

To characterize cancer treatment during pregnancy and maternal and infant outcomes among women with cancer identified during pregnancy in Japan.

**Methods:**

In this descriptive cohort study, we used mother‐infant linkage data from the JMDC claims database from 2005 to 2024. Live‐birth pregnancies were considered eligible when a malignant neoplasm (ICD‐10 C00–C97) was recorded between the estimated pregnancy onset and delivery. Exposure was defined as at least one anticancer agent prescribed between the diagnosis and delivery; surgery and radiation therapy were also identified. The outcomes were preterm delivery and major congenital malformation at 6 months of age. Pregnancies without a diagnosis of malignant neoplasms constituted the internal reference group.

**Results:**

Of the 500 pregnancies with cancer identified during pregnancy, 16 (3.2%) had an anticancer agent prescribed, 46 (9.2%) underwent surgery for malignant tumors, and none underwent radiation therapy. Cancer was identified in the second trimester in 10 of the 16 pregnancies with exposure (62.5%) and in 128 of the 484 pregnancies without exposure (26.4%). All eight pregnancies with breast cancer received an anthracycline with cyclophosphamide. The frequencies in the reference group (*n* = 236 002), without exposure, and with exposure were 12.0%, 20.9%, and 37.5% for preterm delivery and 6.1%, 9.1%, and 12.5% for major congenital malformation, respectively.

**Conclusions:**

Cancer identified during pregnancy was treated in a minority of women, and anticancer agents were most often prescribed when cancer was identified in the second trimester. Outcomes were recorded more often in pregnancies with cancer but cannot be attributed to anticancer agent exposure.

## Key Points

- Assembling the cohort from cancer diagnoses, rather than from the treatment received, kept untreated pregnancies in the denominator, facilitating the description of the proportion treated.
- Of the 500 pregnancies with cancer identified, 16 were prescribed an anticancer agent, 46 underwent malignant tumor surgery, and none received radiation therapy.
- Anticancer agents were prescribed most often when cancer was identified in the second trimester.
- An anthracycline with cyclophosphamide was prescribed to all eight pregnancies with breast cancer.
- Adverse maternal and infant outcomes were recorded more often than in pregnancies without cancer; however, they cannot be attributed to exposure to anticancer agents.

## Plain Language Summary

When a woman is diagnosed with cancer during pregnancy, her doctors have to weigh treating the cancer against the risk of harm to the developing baby. Most knowledge about how these women are treated is from registries and case series. Therefore, how often treatment is provided in routine care remains unclear.

We used health insurance records from Japan, which allowed us to connect maternal records to those of the corresponding infants. We identified 500 pregnancies in which cancer was diagnosed before delivery. Anticancer agents were administered in 16 of these pregnancies (3.2%); cancer surgery was performed in 46 pregnancies (9.2%), and no pregnancies underwent radiation therapy. Women who received anticancer agents were usually those in whom cancer was detected in the second trimester. The drug combinations matched the guidelines recommended at the time.

Problems for mothers and babies, such as early birth and admission to newborn intensive care, were recorded more often in pregnancies with cancer than in those without and more often when anticancer agents were administered. Because we only described what happened in these pregnancies, these differences are not direct evidence that the agents caused them.

## 1 Introduction

Cancer treatment during pregnancy requires balancing maternal benefit against potential fetal harm while coordinating treatment with delivery. Because pregnant women are generally excluded from oncology trials, evidence largely comes from case reports, single‐center series, and expert consensus [1]. Guidelines recommend standard oncological care when regimens are acceptable after the first trimester [2], but how these recommendations are reflected in routine practice at the population level remains unclear.

The International Network on Cancer, Infertility and Pregnancy reported treatment and obstetric and neonatal outcomes for 1170 women registered at 37 centers over 20 years [3]. Treatment was described by drug class and tumor type without examining gestational exposure timing in relation to outcomes. A recent systematic review pooled maternal and neonatal outcomes in pregnancy‐associated cancer without describing cancer treatment [4].

In Japan, a claims‐based case series identified 13 women through anticancer prescriptions during pregnancy, excluding untreated pregnancies [5]. Identifying pregnancies through cancer diagnosis enables mother‐infant linkage, captures both treated and untreated pregnancies, and allows follow‐up of infant diagnoses after birth. Major congenital malformation (MCM) has previously been reported in a nonmalignant obstetric population within the same database [6, 7].

Therefore, we conducted a descriptive claims‐based study with mother‐infant linkage to characterize cancer treatment during pregnancy in Japan and the associated maternal and infant outcomes, alongside those of pregnancies without a diagnosis of malignant neoplasm in the same database.

## 2 Methods

### 2.1 Study Design and Data Source

We conducted a descriptive cohort study using a mother‐infant linked extract of the JMDC claims database, covering Japanese employment‐based health insurance societies (January 2005–December 2024) [8, 9]. A family identifier is assigned to the subscriber and all enrolled dependents, allowing mother‐infant linkage when both are enrolled under the same subscriber. Pregnancy onset and delivery date were estimated from gestational weeks recorded with obstetric diagnoses, following a previously developed algorithm [6]. Universal health insurance in Japan covers both inpatient and outpatient care, captured in these claims records. We followed the RECORD‐PE statement, an extension of RECORD and STROBE for pharmacoepidemiology [10].

### 2.2 Cohort Construction

The cohort construction is shown in Figure 1. Pregnancies were eligible when both the mother and infant had a nonmissing family identifier; gestational weeks were recorded to estimate pregnancy onset; the estimated gestational age was 22–42 weeks; the woman was followed from 3 months before pregnancy onset through delivery; and the child was followed for at least 6 months after birth or died within that period. Pregnancies were excluded when the infant had a genetic disorder or teratogenic syndrome or when the mother had an infection known to cause malformations. Because multiple gestations received more than one pregnancy identifier, records were consolidated at the mother‐infant linkage level and treated as a single pregnancy, while each linked infant was retained. This resulted in 241 891 pregnancies. The cohort was restricted to live births because infant outcomes were ascertained from the infant's own claims records.

**FIGURE 1:**
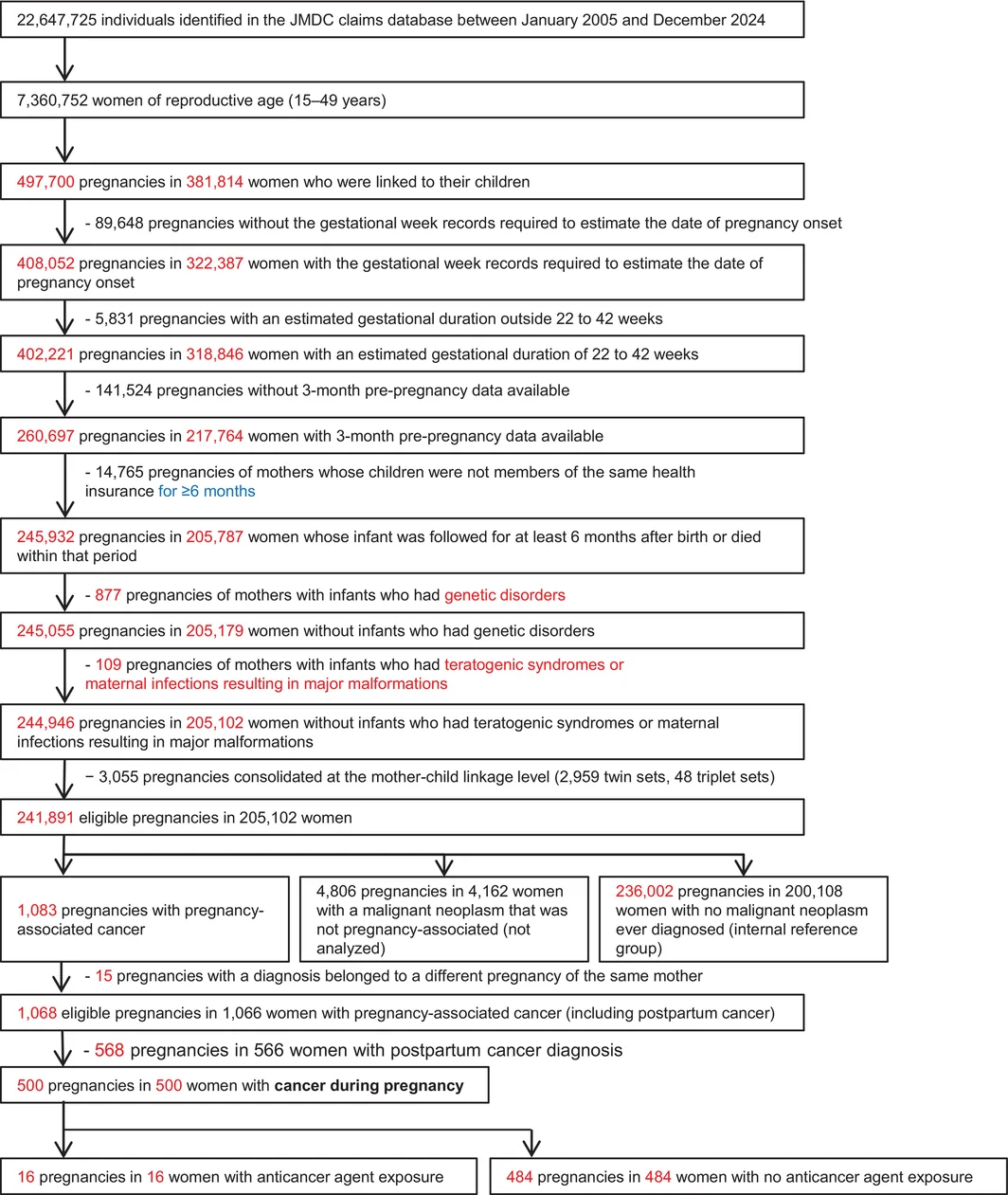
Construction of the study cohort and the internal reference group. Eligible pregnancies were assigned to one of three groups. Pregnancy‐associated cancer was defined as a malignant neoplasm recorded between the estimated pregnancy onset and 1 year after delivery. Pregnancies in women who had a malignant neoplasm recorded at some point in their claims records, but not within this window for the pregnancy concerned, are shown as pregnancies with a malignant neoplasm that was not pregnancy‐associated. These pregnancies were not analyzed and were not included in the internal reference group, which was restricted to women with no malignant neoplasm diagnosis at any point in their claims records.

### 2.3 Cohort Definition and Internal Reference Group

Cancer was identified by malignant neoplasm diagnoses (International Classification of Diseases, 10th revision [ICD‐10] C00–C97), excluding those recorded as suspected. In situ, benign, and neoplasms of uncertain behavior (D00–D48) were not counted. Pregnancy‐associated cancer was defined as a malignant neoplasm whose recorded diagnosis was between the estimated pregnancy onset and 1 year after delivery (*n* = 1083) [11, 12]. Fifteen pregnancies were excluded because the qualifying diagnosis belonged to another pregnancy of the same woman, leaving 1068 pregnancies. The analytical cohort was restricted to 500 pregnancies with a diagnosis recorded before delivery; 568 pregnancies with a postpartum diagnosis were not analyzed further. Pregnancies with more than one primary site were retained; therefore, the total number of cancer types exceeded the number of pregnancies. The internal reference group comprised 236 002 pregnancies in women with no malignant neoplasm diagnoses in their claims records. This group served as a background benchmark rather than a comparator for causal inference. Pregnancies were not excluded when the same cancer was recorded before pregnancy onset, because the objective was to describe treatment during pregnancy, irrespective of whether the disease was newly identified or preexisting.

### 2.4 Exposure Definition

Anticancer agents were identified as all drugs classified under EPhMRA anatomical classification groups L01 and L02. Pregnancies were classified as exposed when at least one anticancer agent was prescribed between cancer diagnosis and delivery (*n* = 16), and as unexposed otherwise (*n* = 484). Treatment during pregnancy was described for all recorded modalities, and outcomes were presented by anticancer agent exposure because these agents are the only modality that exposes the fetus to a drug. Surgery was not considered a separate exposure because the two modalities were not mutually exclusive; it was reported for each exposure group. The window began at cancer diagnosis because anticancer agents may also be prescribed for other indications. The gestational week at the first prescription was calculated from the estimated pregnancy onset and classified by trimester. Trimesters were defined as pregnancy onset through Week 13, Weeks 14 to 27, and Week 28 onward [6]. Regimens were assigned based on the combination of anticancer agents prescribed between diagnosis and delivery; the labels therefore represent observed combinations rather than confirmed regimens.

### 2.5 Cancer Treatment and Maternal Outcomes

Malignant tumor surgery, cervical procedures, and radiation therapy were identified using procedure codes. Surgery and radiation therapy during pregnancy were assessed from pregnancy onset to delivery; prior radiation therapy was that recorded before pregnancy onset. Maternal outcomes included cesarean delivery, high‐risk pregnancy and high‐risk delivery management fees, preterm delivery (gestational duration < 259 days), and severe maternal morbidity (SMM), defined as the presence of at least one of the seven indicators adapted from the CDC 21‐indicator index [13] and ascertainable in claims data: blood transfusion, peripartum hysterectomy, intensive care unit (ICU) admission, mechanical ventilation, sepsis, venous thromboembolism, and eclampsia.

### 2.6 Infant Outcomes

Infant outcomes were ascertained from the infant's claims records. Neonatal ICU and perinatal ICU admissions were defined based on the corresponding management fees and assessed from delivery to 90 days after delivery. MCM was defined using the Quebec Pregnancy Cohort algorithm applied to ICD‐10 codes Q00–Q89, excluding designated minor anomalies, with an ascertainment period from birth to 6 months of age [14, 15]. Excluded codes and results of applying the algorithm to the Japanese Disease Code master are provided in Table S2. In a Japanese validation study, the positive predictive value was 91.5% for MCM and 90.7% for any congenital malformation [16]. Chromosomal abnormalities (Q90–Q99) were not included [7]. Infants who died during an ascertainment period were classified using diagnoses recorded up to death.

### 2.7 Other Variables

Maternal age at pregnancy onset and delivery, calendar year of delivery, multiple gestations, comorbidities, and previous deliveries were described for each group. Comorbidities were identified from ICD‐10 codes recorded 3 months before pregnancy onset through delivery using a code set adapted from a previous work in this database [17]. Previous deliveries were counted within the database observation period and did not represent lifetime parity. Suspected diagnoses were excluded from all diagnosis‐based variables and outcomes.

### 2.8 Statistical Analysis

This was a descriptive analysis. Categorical variables are reported as counts and percentages, and continuous variables as medians with interquartile ranges (IQRs). No hypothesis testing, matching, or adjusted estimation was performed because the 16 pregnancies with exposure were too few. For sensitivity analyses, MCM was tabulated at 3 and 12 months, and any congenital malformation, defined without excluding minor anomalies, at 3, 6, and 12 months. For the 12‐month period, the cohort was restricted to infants followed for 12 months or who died within that period; therefore, the denominators differed. No data were imputed. Radiation therapy records without a date were reported separately as having unknown timing. Analyses were performed using SAS version 9.4 (SAS Institute, Cary, North Carolina, USA).

## 3 Results

### 3.1 Cohort Construction

Of the 241 891 eligible pregnancies, 236 002 among 200 108 women had no malignant neoplasm diagnosis at any point in the claims records, constituting the internal reference group (Figure 1). Pregnancy‐associated cancer was identified in 1068 pregnancies among 1066 women; 568 were diagnosed after delivery, leaving 500 with cancer identified during pregnancy. An anticancer agent was prescribed between cancer diagnosis and delivery in 16 (3.2%) and not in 484 (96.8%) pregnancies. These 500 pregnancies included 12 multiple gestations and were linked to 511 infants; one multiple gestation was linked to only one infant.

### 3.2 Characteristics of the Study Population

Median maternal age at pregnancy onset was 32 years (IQR, 29–35), 34 (30–37), and 32.5 (30–37) in the reference group and cancer groups without and with anticancer agent exposure, respectively. Other characteristics and comorbidities are shown in Tables 1 and S3.

**TABLE 1:** Characteristics of women with cancer during pregnancy, by anticancer agent exposure, with an internal reference group of pregnancies without cancer (*n* = 236 002).

Characteristic	Reference group (*n* = 236 002)	No anticancer agent exposure (*n* = 484)	Anticancer agent exposure (*n* = 16)
Maternal age at pregnancy onset, years, median (IQR)	32 (29–35)	34 (30–37)	32.5 (30–37)
Maternal age at delivery, years, median (IQR)	33 (29–36)	35 (31–38)	33 (31–38)
Year of delivery, *n* (%)			
2005–2010	5642 (2.4%)	12 (2.5%)	0 (0%)
2011–2015	38 854 (16.5%)	91 (18.8%)	1 (6.3%)
2016–2020	108 108 (45.8%)	198 (40.9%)	7 (43.8%)
2021–2024	83 398 (35.3%)	183 (37.8%)	8 (50.0%)
Multiple gestation, *n* (%)	2914 (1.2%)	11 (2.3%)	1 (6.3%)
Any comorbidity^a^, *n* (%)	60 496 (25.6%)	192 (39.7%)	5 (31.3%)
Clinical characteristics of women with cancer during pregnancy			
Gestational week at cancer diagnosis, weeks, median (IQR)	—	16 (8–29)	19 (11–26.5)
Days from pregnancy onset to cancer diagnosis, days, median (IQR)	—	112 (60–207)	135.5 (81–187.5)
Days from cancer diagnosis to delivery, days, median (IQR)	—	153 (54.5–209)	111.5 (63–166.5)
Gestational timing at cancer diagnosis, *n* (%)			
First trimester	—	214 (44.2%)	4 (25.0%)
Second trimester	—	128 (26.4%)	10 (62.5%)
Third trimester	—	142 (29.3%)	2 (12.5%)
Previous deliveries recorded in the database^b^, *n* (%)			
0	200 108 (84.8%)	442 (91.3%)	14 (87.5%)
1	33 049 (14.0%)	37 (7.6%)	2 (12.5%)
2	2647 (1.1%)	5 (1.0%)	0 (0%)
3	175 (0.1%)	0 (0%)	0 (0%)
4	21 (< 0.1%)	0 (0%)	0 (0%)
5	2 (< 0.1%)	0 (0%)	0 (0%)
Primary cancer site^c^, *n* (%)			
Thyroid cancer	—	95 (19.6%)	0 (0%)
Cervical cancer	—	92 (19.0%)	1 (6.3%)
Breast cancer	—	64 (13.2%)	8 (50.0%)
Adult T‐cell leukemia/lymphoma	—	50 (10.3%)	0 (0%)
Ovarian cancer	—	37 (7.6%)	1 (6.3%)
Non‐Hodgkin lymphoma	—	20 (4.1%)	1 (6.3%)
Colorectal cancer	—	16 (3.3%)	0 (0%)
Cancer of unknown primary	—	14 (2.9%)	0 (0%)
Endometrial cancer	—	13 (2.7%)	0 (0%)
Gastric cancer	—	10 (2.1%)	2 (12.5%)
Liver cancer	—	8 (1.7%)	0 (0%)
Acute myeloid leukemia	—	7 (1.4%)	1 (6.3%)
Head and neck cancer	—	8 (1.7%)	0 (0%)
Bone and soft tissue tumors	—	6 (1.2%)	0 (0%)
Central nervous system tumors	—	5 (1.0%)	0 (0%)
Lung cancer	—	5 (1.0%)	0 (0%)
Chronic myeloid leukemia	—	4 (0.8%)	1 (6.3%)
Multiple myeloma and plasma cell neoplasms	—	4 (0.8%)	0 (0%)
Other hematologic malignancies	—	3 (0.6%)	0 (0%)
Other endocrine tumors	—	3 (0.6%)	0 (0%)
Acute lymphoblastic leukemia	—	2 (0.4%)	1 (6.3%)
Skin cancer	—	3 (0.6%)	0 (0%)
Gestational trophoblastic neoplasia	—	3 (0.6%)	0 (0%)
Other female genital cancers	—	2 (0.4%)	0 (0%)
Hodgkin lymphoma	—	2 (0.4%)	0 (0%)
Uterine cancer, unspecified site	—	2 (0.4%)	0 (0%)
Metastatic malignancy	—	2 (0.4%)	0 (0%)
Leukemia, unspecified	—	2 (0.4%)	0 (0%)
Urinary tract cancer	—	2 (0.4%)	0 (0%)
Pancreatic cancer	—	2 (0.4%)	0 (0%)
Other digestive cancers	—	1 (0.2%)	0 (0%)
Malignant melanoma	—	1 (0.2%)	0 (0%)
Acute leukemia, unspecified	—	1 (0.2%)	0 (0%)
Esophageal cancer	—	1 (0.2%)	0 (0%)
Chronic‐phase chronic myeloid leukemia	—	1 (0.2%)	0 (0%)

*Note:* Percentages were calculated within each group (reference group, *n* = 236 002; no anticancer agent exposure, *n* = 484; anticancer agent exposure, *n* = 16). The reference group comprised pregnancies among women with no malignant neoplasm (ICD‐10 C00‐C97) diagnosis recorded at any point in their claims history; clinical characteristics specific to the cancer cohort (gestational timing at diagnosis and primary cancer site) do not apply to the reference group (indicated by —).

^a^ Comorbidities were defined using ICD‐10 diagnosis codes recorded from 3 months before pregnancy onset through delivery, adapted from a preexisting comorbidity code set (Ishikawa et al., Pregnancy Hypertens. 2023); individual comorbidity categories are reported in Table S3.

^b^ The distribution of previous deliveries is not directly comparable across groups because the unit of observation differs. The reference group contributed all eligible pregnancies per woman (236 002 pregnancies among 200 108 women), whereas each woman in the cancer cohort contributed a single pregnancy. Only the first pregnancy observed for a woman can have no previous delivery recorded, so a group that also contributes second and later pregnancies shows a lower proportion with no previous delivery by construction.

^c^ Percentages may not sum to 100% because multiple primary cancer sites were allowed for a single pregnancy.

### 3.3 Primary Cancer Sites and Timing of Diagnosis

Among pregnancies without anticancer agent exposure, the most frequent primary sites were the thyroid (95, 19.6%), cervical (92, 19.0%), breast (64, 13.2%), and adult T‐cell leukemia/lymphoma (50, 10.3%). Breast cancer accounted for 8 of 16 (50.0%) and gastric cancer for 2 (12.5%); no pregnancy with thyroid cancer had exposure. Median gestational week at diagnosis was 16 (IQR 8–29) without exposure, and 19 (IQR 11–26.5) with exposure, and the median interval from diagnosis to delivery was 153 days (IQR 54.5–209) and 111.5 days (IQR 63–166.5). Cancer was diagnosed in the first trimester in 214 pregnancies without exposure (44.2%) and 4 with exposure (25.0%), in the second in 128 (26.4%) and 10 (62.5%), and in the third in 142 (29.3%) and 2 (12.5%), respectively.

### 3.4 Cancer Treatment During Pregnancy

Malignant tumor surgery was performed during pregnancy in 46 pregnancies (9.2%): 39 without anticancer agent exposure (8.1%) and 7 with exposure (43.8%; Table 2). Cervical procedures were performed in 10 (2.0% of the total cohort), all without anticancer agent exposure. Radiation therapy was not recorded during pregnancy. It was recorded before pregnancy onset in 18 pregnancies (3.6%), and its timing could not be determined in 3 (0.6%). At least one of these treatments was recorded in 55 pregnancies (11.0%). Counting each pregnancy once, 39 (7.8%) had surgery only, nine (1.8%) an anticancer agent only, seven (1.4%) both, and 445 (89.0%) neither. Characteristics and outcomes by these categories are shown in Table S5.

**TABLE 2:** Cancer treatment during pregnancy among women with cancer identified during pregnancy (*n* = 500) and details of anticancer agent prescriptions (*n* = 16).

Characteristic	Total (*n* = 500)	No anticancer agent exposure (*n* = 484)	Anticancer agent exposure (*n* = 16)
Cancer treatment during pregnancy, *n* (%)			
Anticancer agent prescription	16 (3.2%)	—	—
Malignant tumor surgery	46 (9.2%)	39 (8.1%)	7 (43.8%)
Cervical procedure (K867 series)^a^	10 (2.0%)	10 (2.1%)	0 (0%)
Radiation therapy during pregnancy	0 (0%)	0 (0%)	0 (0%)
Prior radiation therapy history	18 (3.6%)	18 (3.7%)	0 (0%)
Radiation history, timing unknown	3 (0.6%)	2 (0.4%)	1 (6.3%)
Details of anticancer agent prescriptions (*n* = 16)			
Gestational week at first anticancer agent prescription, weeks, median (IQR)	—	—	24.5 (20.5–29.0)
Days from cancer diagnosis to first anticancer agent prescription, days, median (IQR)	—	—	29.5 (10.0–64.5)
Trimester at first anticancer agent prescription, *n* (%)			
First trimester	—	—	1 (6.3%)
Second trimester	—	—	8 (50.0%)
Third trimester	—	—	7 (43.8%)

*Note:* Percentages in the upper section were calculated within each group (total, *n* = 500; no anticancer agent exposure, *n* = 484; anticancer agent exposure, *n* = 16); percentages in the lower section were calculated among the 16 pregnancies with anticancer agent prescriptions. The anticancer agent prescription row defines the exposure groups and is therefore presented for the total cohort only.

^a^ Cervical procedures (K867 series) include both invasive cancer treatment and procedures for noninvasive cervical lesions; all 10 cases occurred among pregnancies with cervical cancer as the primary site. Malignant tumor surgery and radiation therapy during pregnancy were assessed from pregnancy onset to delivery; prior radiation therapy history refers to radiation therapy recorded before pregnancy onset. For three additional pregnancies with a history of radiation therapy, the date could not be determined and timing relative to pregnancy onset could not be classified.

### 3.5 Details of Anticancer Agent Prescriptions

Among the 16 pregnancies with anticancer agent exposure, the median gestational week at the first prescription was 24.5 (IQR 20.5–29.0). The first prescription was within the first trimester in one pregnancy (6.3%), the second in eight (50.0%), and the third in seven (43.8%; Table 2). The median interval from diagnosis to the first prescription was 29.5 days (IQR 10.0–64.5) overall. It was 102.5 days in the four pregnancies diagnosed in the first trimester, 21 days in the 10 diagnosed in the second, and 3.5 days in the two diagnosed in the third. Three of the four pregnancies diagnosed in the first trimester began treatment in the second. All eight pregnancies with breast cancer received an anthracycline with cyclophosphamide (doxorubicin in seven, epirubicin in one) but no taxane; a taxane was prescribed in three pregnancies with gastric, cervical, or ovarian cancer. Surgery preceded the first prescription in six of the seven pregnancies in which both were recorded (Table S1).

### 3.6 Maternal Outcomes

In the reference group and cancer groups without and with anticancer agent exposure, respectively, preterm delivery was recorded in 12.0%, 20.9%, and 37.5%; SMM in 4.2%, 9.5%, and 25.0%; and cesarean delivery in 20.4%, 32.0%, and 31.3%. Management fees and individual SMM components are reported in Table 3.

**TABLE 3:** Maternal outcomes among women with cancer during pregnancy, by anticancer agent exposure, with an internal reference group of pregnancies without cancer (*n* = 236 002).

Outcome, *n* (%)	Reference group (*n* = 236 002)	No anticancer agent exposure (*n* = 484)	Anticancer agent exposure (*n* = 16)
Delivery outcomes			
Cesarean delivery	48 042 (20.4%)	155 (32.0%)	5 (31.3%)
High‐risk pregnancy management fee	11 621 (4.9%)	77 (15.9%)	4 (25.0%)
High‐risk delivery management fee	12 083 (5.1%)	65 (13.4%)	4 (25.0%)
Preterm delivery (< 259 days of gestation)	28 288 (12.0%)	101 (20.9%)	6 (37.5%)
Severe maternal morbidity components			
Blood transfusion	3772 (1.6%)	16 (3.3%)	2 (12.5%)
Peripartum hysterectomy	88 (< 0.1%)	0 (0%)	0 (0%)
ICU admission	392 (0.2%)	6 (1.2%)	0 (0%)
Mechanical ventilation	112 (< 0.1%)	0 (0%)	0 (0%)
Sepsis	2092 (0.9%)	8 (1.7%)	1 (6.3%)
Venous thromboembolism	4096 (1.7%)	22 (4.5%)	1 (6.3%)
Eclampsia	380 (0.2%)	0 (0%)	0 (0%)
Any severe maternal morbidity^a^	10 027 (4.2%)	46 (9.5%)	4 (25.0%)

^a^ Percentages were calculated within each group (reference group, *n* = 236 002; no anticancer agent exposure, *n* = 484; anticancer agent exposure, *n* = 16). The reference group comprised pregnancies among women with no malignant neoplasm (ICD‐10 C00‐C97) diagnosis recorded at any point in their claims history. Ascertainment windows: cesarean delivery, pregnancy onset to delivery; high‐risk pregnancy management fee (A236‐2), pregnancy onset to delivery; high‐risk delivery management fee (A237), pregnancy onset to 14 days after delivery. Severe maternal morbidity (SMM) was defined as the presence of at least 1 of the following: blood transfusion, peripartum hysterectomy, ICU admission, mechanical ventilation, sepsis, venous thromboembolism, or eclampsia, adapted from the CDC's 21‐indicator severe maternal morbidity index (Callaghan et al., Obstet Gynecol 2012) and restricted to indicators ascertainable in JMDC claims data and applied identically across all groups. Blood transfusion, ICU admission, and mechanical ventilation were assessed within ±7 days of delivery; peripartum hysterectomy, pregnancy onset to 14 days after delivery; sepsis, venous thromboembolism, and eclampsia, from pregnancy onset to 42 days after delivery, consistent with the ICD‐10 definition of the puerperium. ICU admission did not follow the same order as other SMM components across groups. Among women with cancer during pregnancy, malignant tumor surgery during pregnancy was more frequent in the anticancer agent exposure group (7/16, 43.8%) than in the no anticancer agent exposure group (39/484, 8.1%); the exposure group therefore included pregnancies managed with both surgery and anticancer agents, and the observed differences cannot be attributed to anticancer agent exposure alone. Formal statistical testing was not performed; these are descriptive comparisons only.

### 3.7 Infant Outcomes

Neonatal ICU admission was recorded in 3.2% of the reference group, 5.6% without anticancer agent exposure, and 31.3% with anticancer agent exposure, while perinatal ICU admission was recorded in 4.8%, 13.0%, and 37.5%, respectively (Table 4). Admission to either unit was recorded in 15 918 (6.7%), 79 (16.3%), and 8 (50.0%) pregnancies, respectively. MCM was recorded in 14 288 (6.1%), 44 (9.1%), and 2 (12.5%) pregnancies, respectively. The two malformations in the exposed group were hypertrophic pyloric stenosis (Q40.0), recorded within 1 month of birth, and pulmonary artery stenosis (Q25.6), recorded between 3 and 6 months. With ascertainment periods of 3 and 12 months, MCM frequencies were 4.1%, 5.8%, and 6.3% and 7.2%, 11.1%, and 12.5%, respectively. The frequencies of any congenital malformation at 6 months were 9.8%, 14.9%, and 12.5%, respectively (Table S4).

**TABLE 4:** Infant outcomes among pregnancies of women with cancer during pregnancy, by anticancer agent exposure, with an internal reference group of pregnancies without cancer (*n* = 236 002).

Outcome, *n* (%)	Reference group (*n* = 236 002)	No anticancer agent exposure (*n* = 484)	Anticancer agent exposure (*n* = 16)
Neonatal ICU admission	7493 (3.2%)	27 (5.6%)	5 (31.3%)
Perinatal ICU admission	11 411 (4.8%)	63 (13.0%)	6 (37.5%)
Any neonatal or perinatal ICU admission^a^	15 918 (6.7%)	79 (16.3%)	8 (50.0%)
Major congenital malformation^b^	14 288 (6.1%)	44 (9.1%)	2 (12.5%)

^a^ Outcomes were assessed at the pregnancy level; for multiple gestations, a pregnancy was classified as affected if any linked infant met the outcome criterion. Percentages were calculated within each group (reference group, *n* = 236 002; no anticancer agent exposure, *n* = 484; anticancer agent exposure, *n* = 16). Neonatal ICU admission was defined by neonatal intensive care unit management fees (A302, A302‐2); perinatal ICU admission was defined by comprehensive perinatal intensive care unit management fees (A303, A303‐2), billed under the infant's own claims record. Both were assessed from the date of delivery to 90 days after delivery. For 1 multiple gestation in the no anticancer agent exposure group, only 1 of the linked infants could be identified in the database; infant outcomes for this pregnancy were therefore ascertained from the single linked infant.

^b^ Major congenital malformations were identified using ICD‐10 codes Q00–Q89 after excluding codes designated as minor anomalies according to the Quebec Pregnancy Cohort algorithm (Table S2). Chromosomal abnormalities (Q90–Q99) were not included. Malformations were ascertained from birth to 6 months of age. Infants who died before the end of the ascertainment period were classified using diagnoses recorded up to the date of death. Results using ascertainment windows of 3 and 12 months, and results for any congenital malformation without exclusion of minor anomalies, are presented in Table S4. The frequency observed in the reference group (6.1%) is consistent with a previous report from the same database (6.96%; Ishikawa et al., Birth Defects Res 2023). Formal statistical testing was not performed; these are descriptive comparisons only.

## 4 Discussion

Treatment during pregnancy was uncommon and selective. The main feature distinguishing pregnancies in which an anticancer agent was prescribed was the trimester of diagnosis. At least one cancer‐directed treatment was recorded in 55 of 500 pregnancies (11.0%), compared with 779 of 1170 women (67%) treated during pregnancy in the largest international case series, in which surgery was recorded in 39% and chemotherapy in 37% [3]. These women registered voluntarily at 37 participating centers, which largely selected women for whom antenatal treatment was already under consideration. Identifying pregnancies through cancer diagnosis places treated and untreated pregnancies within the same denominator. Many cancers were managed expectantly, most notably thyroid cancer, which was recorded in 95 of the 500 pregnancies, and was never associated with anticancer agent exposure. International guidance states that chemotherapy should not be given during the first trimester, the period of organogenesis, and considers administration from the second trimester onward acceptable [1]. The timing observed here was consistent with this guidance, and the intervals between diagnosis and first prescription appeared to reflect the point in pregnancy at which cancer was identified rather than a uniform waiting period.

The observed regimens were largely those recommended after the first trimester. All eight pregnancies with breast cancer received an anthracycline with cyclophosphamide and no taxane. This combination is endorsed by international consensus [18] and the Japanese guideline on pregnancy and childbirth in breast cancer [19], which did not present taxanes as an option during pregnancy until the 2026 edition [20]. All pregnancies included herein were delivered by 2024; therefore, the absence of taxanes was consistent with the guidance in force during the study period. For gynecological cancers, a platinum agent with a taxane is recommended from the second trimester [21], and this combination was prescribed in 1 of 93 pregnancies with cervical cancer and in 1 of 38 pregnancies with ovarian cancer.

Three prescription patterns differed from current guidelines. One pregnancy with breast cancer received doxorubicin with cyclophosphamide at an estimated 11 weeks, whereas the other three pregnancies diagnosed in the first trimester began treatment in the second trimester (Table S1). Because gestational week was estimated, the trimester assigned to this prescription was uncertain. Two pregnancies with gastric cancer received S‐1, contraindicated during pregnancy in Japan; in one, it was prescribed as monotherapy at 19 weeks. One pregnancy with chronic myeloid leukemia received mercaptopurine alone at 32 weeks. Although this agent has an approved indication for this disease in Japan, guidance for pregnant patients favors interferon and does not describe mercaptopurine [22]. These prescriptions were few, but fell outside the treatment approaches described in the relevant guidance.

Adverse maternal and infant outcomes were recorded more often in pregnancies with cancer than in the reference group, and more often with anticancer agent exposure. This pattern was observed for SMM, preterm delivery, and neonatal or perinatal ICU admission (Tables 3 and 4). Similarly, a population‐based study in California reported greater severe maternal and neonatal morbidity with cancer diagnosed during pregnancy [23]. These differences cannot be attributed to anticancer agent exposure because treatment intensity, cancer severity, and stage could not be separated, and 7 of the 16 pregnancies with exposure also underwent surgery. The pattern was not uniform; cesarean delivery was more frequent in pregnancies with cancer than in the reference group (32.0% vs. 20.4%), but was not more frequent with anticancer agent exposure (31.3%).

MCM in the reference group, 6.1% at 6 months, was similar to the 6.96% previously recorded in the same database using the same algorithm [7] but exceeded birth prevalence reported by malformation registries, which use active ascertainment rather than diagnoses recorded in claims [14]. These values represent detection frequencies rather than birth prevalence. Within this framework, the two malformations in pregnancies with anticancer agent exposure involved different organ systems and were too few to indicate a pattern. Any congenital malformation was also recorded less often with exposure than without exposure at every ascertainment period. In both pregnancies with anticancer agent exposure and a recorded malformation, the first prescription was given after organogenesis, at 24 and 33 weeks. MCM was also recorded more often in pregnancies with cancer without exposure than in the reference group; therefore, the ordering across the three groups was not specific to anticancer agent exposure.

These findings describe treatment during pregnancy at the population level rather than indicating which treatment should be selected. They provide a reference point for counseling that referral‐based case series cannot provide and show that prescriptions outside pregnancy‐specific guidance can be identified in claims data, making such prescribing amenable to routine medication safety review.

### 4.1 Strengths and Limitations

The cohort was assembled from cancer diagnoses rather than treatment received; therefore, pregnancies managed without anticancer agents remained in the denominator, allowing the proportion treated to be described. Mother‐infant linkage allowed infant outcomes to be ascertained from the infant's own claims throughout the 6‐month ascertainment period. The internal reference group was drawn from the same database and study period, and all outcomes were measured similarly across the three groups.

The principal limitation was the size of the group with anticancer agent exposure. Among the 16 exposed pregnancies, a single pregnancy changed a frequency by 6.3 percentage points. Treatment intensity could not be separated from exposure because 7 of these 16 pregnancies also underwent surgery, while stage, histology, and treatment intent were not recorded in claims; therefore, confounding by disease severity remained possible. The diagnosis date was defined as the first record within the study window, so pregnancies in which previously known cancer was re‐recorded during pregnancy were included. This applied to 1 of the 16 pregnancies with anticancer agent exposure and 130 of the 484 without exposure (26.9%). The groups also differed in composition because each woman in the reference group could contribute multiple pregnancies, whereas the cancer cohort contributed one pregnancy per woman, potentially affecting outcomes related to previous deliveries, such as cesarean delivery. Exposure was defined based on claims, and for the three pregnancies in which an oral agent was prescribed, actual medication intake could not be confirmed; dose, treatment cycles, and duration also were not characterized. As the cohort required linkage to a live‐born infant, miscarriage, stillbirth, and termination were not captured. Pregnancies ending in loss or termination are enriched for fetal malformations; therefore, fetuses with such malformations were absent from all three groups. If cancer or its treatment increased the probability of pregnancy loss, this exclusion could be greater in the exposed group and could attenuate rather than produce the higher frequency observed in that group. Birth weight and Apgar score were not captured; therefore, small for gestational age, an outcome associated with antenatal chemotherapy, could not be examined [3]. The SMM indicator was not comparable with published indices. Finally, the source population comprised enrollees of employment‐based insurance societies; therefore, the distribution of cancer types may differ from that in other settings. Linkage also required mother and infant to be enrolled under the same subscriber; therefore, pregnancies in which the infant was enrolled separately were not captured. This arrangement is unlikely to depend on the cancer diagnosis or treatment, although this could not be examined.

## 5 Conclusion

Cancer identified during pregnancy was treated during pregnancy in a few women, and anticancer agents were most often prescribed when cancer was identified in the second trimester. Maternal and infant adverse outcomes were recorded more often than in pregnancies without a diagnosis of malignant neoplasm, although a descriptive design cannot establish whether these frequencies reflect anticancer agent exposure; therefore, comparative studies are needed.

## Author Contributions

M.T. conceived the study, designed the analysis, developed and executed the analysis code, interpreted the results, and drafted the manuscript. T.I. and T.S. contributed to the algorithms and outcome definitions applied to the claims data and reviewed the analysis code. S.H. supervised the study and critically revised the manuscript. T.O. performed data extraction, supervised the study design and analysis, and critically revised the manuscript. All authors approved the final version of the manuscript.

## Funding

This work was supported by JSPS KAKENHI Grant Number 23K14428 (Grant‐in‐Aid for Early Career Scientists).

## Ethics Statement

The Institutional Review Board of the Tohoku University Graduate School of Medicine approved this study (2024‐1‐178; June 19, 2024). The requirement for informed consent was waived because the database contained anonymized data.

## Conflicts of Interest

T.I. is an employee of Pfizer Research and Development in Japan and is affiliated with Tohoku University. He contributed to this study independently of Pfizer R&D Japan. The other authors declare no conflicts of interest.

## Supporting information


**Table S1:** Details of women with anticancer agent prescriptions between cancer diagnosis and delivery.
**Table S2:** Application of the Quebec Pregnancy Cohort algorithm to the Japanese standardized disease code master, by organ system.
**Table S3:** Individual comorbidity categories among women with cancer during pregnancy, by anticancer agent exposure, with an internal reference group of pregnancies without cancer (*n* = 236 002).
**Table S4:** Congenital malformations by ascertainment period among pregnancies of women with cancer during pregnancy, by anticancer agent exposure, with an internal reference group of pregnancies without cancer.
**Table S5:** Characteristics and outcomes by mutually exclusive treatment category among women with cancer identified during pregnancy (*n* = 500).

## Data Availability

The data that support the findings of this study are available from JMDC Inc. Restrictions apply to the availability of these data, which were used under license for this study. Data are available from https://www.jmdc.co.jp/en/jmdc‐claims‐database/ with the permission of JMDC Inc.
