# Supplementary material for: Cancer Treatment During Pregnancy and Maternal and Infant Outcomes: A Descriptive Cohort Study Using a Japanese Claims Database
Source: Pharmacoepidemiol Drug Saf. 2026 Oct 16;35(11):e70508. doi: 10.1002/pds.70508 (PMC13675649; doi:10.1002/pds.70508)
Supplement: Supplementary file 1 [file PDS-35-e70508-s001.docx]

**Table S1.** Details of women with anticancer agent prescriptions between cancer diagnosis and delivery

| No. | Cancer type | Days from cancer diagnosis to first prescription of anticancer agent | Gestational week at first prescription of anticancer agent | Trimester at first prescription of anticancer agent | Regimen | Malignant tumor surgery, days from first prescription of anticancer agent† | Days from surgery to delivery |
| --- | --- | --- | --- | --- | --- | --- | --- |
| 1 | Breast cancer | 41 | 22 | Second trimester | ADR+CPA | -24 | 130 |
| 2 | Breast cancer | 12 | 29 | Third trimester | ADR+CPA | – | – |
| 3 | Breast cancer | 7 | 28 | Third trimester | ADR+CPA | – | – |
| 4 | Breast cancer | 38 | 29 | Third trimester | ADR+CPA | – | – |
| 5 | Breast cancer | 88 | 20 | Second trimester | ADR+CPA | – | – |
| 6 | Breast cancer | 33 | 11 | First trimester | ADR+CPA | -7 | 146 |
| 7 | Breast cancer | 131 | 24 | Second trimester | EPI+CPA | -49 | 151 |
| 8 | Breast cancer | 111 | 31 | Third trimester | ADR+CPA | -40 | 90 |
| 9 | Gastric cancer | 26 | 25 | Second trimester | S-1+DTX | – | – |
| 10 | Gastric cancer | 117 | 19 | Second trimester | S-1 | -39 | 188 |
| 11 | Cervical cancer | 16 | 18 | Second trimester | CBDCA+PTX | +98† | 0 |
| 12 | Ovarian cancer | 34 | 22 | Second trimester | CBDCA+PTX | – | – |
| 13 | Acute lymphoblastic leukemia | 8 | 21 | Second trimester | DNR+CPA+VCR, MTX+AraC+L-Asp | – | – |
| 14 | Acute myeloid leukemia | 7 | 33 | Third trimester | DNR+AraC | – | – |
| 15 | Chronic myeloid leukemia | 0 | 32 | Third trimester | 6-MP | – | – |
| 16 | Non-Hodgkin lymphoma | 14 | 28 | Third trimester | CHOP | -43 | 85 |

*Regimens were assigned based on anticancer agents prescribed between cancer diagnosis and delivery. ADR, doxorubicin; CPA, cyclophosphamide; EPI, epirubicin; DTX, docetaxel; CBDCA, carboplatin; PTX, paclitaxel; DNR, daunorubicin; VCR, vincristine; MTX, methotrexate; AraC, cytarabine; L-Asp, L-asparaginase; 6-MP, mercaptopurine; CHOP, cyclophosphamide, doxorubicin, vincristine, and prednisone.

†Surgery timing is expressed as days from the first prescription of anticancer agent (negative values indicate surgery preceded the first prescription of anticancer agent; positive values indicate surgery followed it). For No. 11 (cervical cancer), malignant tumor surgery (uterine malignant tumor surgery, K879) was performed on the day of delivery, consistent with radical hysterectomy performed concurrently with cesarean delivery following neoadjuvant chemotherapy.

**Table S2.** Application of the Quebec Pregnancy Cohort algorithm to the Japanese standardized disease code master, by organ system

| **Organ system (ICD-10)** | **Codes assessed** | **Classified as major** | **Excluded as minor, n (%)** |
| --- | --- | --- | --- |
| Nervous system (Q00–Q07) | 96 | 96 | 0 (0%) |
| Eye, ear, face and neck (Q10–Q18) | 135 | 81 | 54 (40.0%) |
| Circulatory system (Q20–Q28) | 254 | 250 | 4 (1.6%) |
| Respiratory system (Q30–Q34) | 57 | 57 | 0 (0%) |
| Cleft lip and cleft palate (Q35–Q37) | 41 | 41 | 0 (0%) |
| Digestive system (Q38–Q45) | 162 | 160 | 2 (1.2%) |
| Genital organs (Q50–Q56) | 92 | 69 | 23 (25.0%) |
| Urinary system (Q60–Q64) | 95 | 95 | 0 (0%) |
| Musculoskeletal system (Q65–Q79) | 291 | 266 | 25 (8.6%) |
| Other congenital malformations (Q80–Q89) | 254 | 156 | 98 (38.6%) |
| **Total** | **1,477** | **1,271** | **206 (14.0%)** |

The Quebec Pregnancy Cohort algorithm (ICD-10 Q00–Q89, excluding codes designated as minor anomalies) was applied to the Japanese standardized disease code master. Of 1,477 codes within Q00–Q89, 1,271 (86.1%) were classified as major congenital malformations and 206 (14.0%) were excluded as minor anomalies. Exclusion was concentrated in the eye, ear, face and neck (40.0%), other congenital malformations (38.6%), and genital organs (25.0%) categories. Chromosomal abnormalities (Q90–Q99) were not included in the algorithm. The validity of this algorithm in Japanese claims data has been reported previously (Ishikawa et al., Pharmacoepidemiol Drug Saf 2021; positive predictive value 91.5% for major congenital malformations). Codes excluded as minor anomalies were Q10; Q16.2; Q17, Q18.0 to Q18.2, and Q18.4 to Q18.9; Q25.0; Q27.0; Q38.1; Q51.5; Q51.6; Q52.0 to Q52.7; Q53; Q66.4 to Q66.6; Q69; Q70; and Q81 to Q84. Q18.3 was retained as a major congenital malformation.

**Table S3.** Individual comorbidity categories among women with cancer during pregnancy, by anticancer agent exposure, with an internal reference group of pregnancies without cancer (n=236,002)

| **Comorbidity, n (%)** | **Reference group (n=236,002)** | **No anticancer agent exposure (n=484)** | **Anticancer agent exposure (n=16)** |
| --- | --- | --- | --- |
| Any comorbidity | 60,496 (25.6%) | 192 (39.7%) | 5 (31.3%) |
| Psychiatric disorders | 15,214 (6.5%) | 59 (12.2%) | 2 (12.5%) |
| Autoimmune disease | 3,349 (1.4%) | 28 (5.8%) | 1 (6.3%) |
| Diabetes mellitus | 6,567 (2.8%) | 49 (10.1%) | 0 (0%) |
| Gestational diabetes | 18,966 (8.0%) | 47 (9.7%) | 3 (18.8%) |
| Hypertension | 6,543 (2.8%) | 21 (4.3%) | 0 (0%) |
| Hypertensive disorders of pregnancy | 18,998 (8.1%) | 39 (8.1%) | 0 (0%) |
| Epilepsy | 1,348 (0.6%) | 11 (2.3%) | 0 (0%) |
| Obesity | 2,318 (1.0%) | 14 (2.9%) | 0 (0%) |
| Dyslipidemia | 5,070 (2.2%) | 35 (7.2%) | 0 (0%) |
| Renal disease | 293 (0.1%) | 4 (0.8%) | 0 (0%) |
| Cardiovascular disease | 3,021 (1.3%) | 27 (5.6%) | 1 (6.3%) |
| Myocardial infarction | 73 (<0.1%) | 0 (0%) | 0 (0%) |
| Congestive heart failure | 1,071 (0.5%) | 12 (2.5%) | 0 (0%) |
| Cerebrovascular disease | 780 (0.3%) | 8 (1.7%) | 0 (0%) |
| Substance dependence | 179 (0.1%) | 1 (0.2%) | 0 (0%) |

Comorbidities were ascertained using ICD-10 diagnosis codes recorded from 3 months before pregnancy onset through delivery. Any comorbidity indicates the presence of at least 1 of the listed categories; individual categories are not mutually exclusive. Psychiatric disorders excluded diagnoses related to alcohol or substance use (ICD-10 F10–F19), which are reported separately as substance dependence. Malignant neoplasm was not included as a comorbidity category because it is definitionally related to cohort eligibility.

**Table S4.** Congenital malformations by ascertainment period among pregnancies of women with cancer during pregnancy, by anticancer agent exposure, with an internal reference group of pregnancies without cancer

| **Outcome and ascertainment period** | **Reference group** | **No anticancer agent exposure** | **Anticancer agent exposure** |
| --- | --- | --- | --- |
| Pregnancies analyzed, n |  |  |  |
| 3 months | 236,002 | 484 | 16 |
| 6 months (main analysis) | 236,002 | 484 | 16 |
| 12 months | 221,807 | 458 | 16 |
| Major congenital malformation, n (%) |  |  |  |
| 3 months | 9,717 (4.1%) | 28 (5.8%) | 1 (6.3%) |
| 6 months (main analysis) | 14,288 (6.1%) | 44 (9.1%) | 2 (12.5%) |
| 12 months | 16,078 (7.2%) | 51 (11.1%) | 2 (12.5%) |
| Any congenital malformation, n (%) |  |  |  |
| 3 months | 15,223 (6.5%) | 52 (10.7%) | 1 (6.3%) |
| 6 months | 23,086 (9.8%) | 72 (14.9%) | 2 (12.5%) |
| 12 months | 27,210 (12.3%) | 87 (19.0%) | 2 (12.5%) |

Major congenital malformations were identified using ICD-10 codes Q00–Q89 after excluding codes designated as minor anomalies according to the Quebec Pregnancy Cohort algorithm (Table S2); any congenital malformation includes all codes within Q00–Q89 without exclusion of minor anomalies. Chromosomal abnormalities (Q90–Q99) were not included. Outcomes were assessed at the pregnancy level; for multiple gestations, a pregnancy was classified as affected if any linked child met the outcome criterion. Infants who died before the end of an ascertainment period were classified using diagnoses recorded up to the date of death. The 6-month ascertainment period is the main analysis reported in Table 4. For the 12-month ascertainment period, the analytic population was restricted to pregnancies in which the child could be followed for 12 months or died within 12 months, which reduced the number of pregnancies analyzed in the reference group and in the group without anticancer agent exposure; all 16 pregnancies with anticancer agent exposure met this requirement. Frequencies across ascertainment periods are therefore not based on identical populations, and comparisons across periods should focus on whether the ordering among groups is preserved rather than on absolute frequencies. Formal statistical testing was not performed; these are descriptive comparisons only.

**Table S5.** Characteristics and outcomes by mutually exclusive treatment category among women with cancer identified during pregnancy (n=500)

| **Characteristic** | **Neither (n=445)** | **Surgery only (n=39)** | **Anticancer agent only (n=9)** | **Both (n=7)** |
| --- | --- | --- | --- | --- |
| **Primary cancer site, n** |  |  |  |  |
| Thyroid | 94 | 1 | 0 | 0 |
| Cervical | 77 | 14 | 0 | 1 |
| Breast | 49 | 15 | 4 | 4 |
| Ovarian | 30 | 5 | 1 | 0 |
| Hematologic malignancies | 96 | 0 | 3 | 1 |
| Other | 99 | 4 | 1 | 1 |
| **Outcomes, n (%)** |  |  |  |  |
| Cesarean delivery | 143 (32.1%) | 12 (30.8%) | 3 (33.3%) | 2 (28.6%) |
| Preterm delivery | 90 (20.2%) | 11 (28.2%) | 3 (33.3%) | 3 (42.9%) |
| Severe maternal morbidity | 40 (9.0%) | 6 (15.4%) | 1 (11.1%) | 3 (42.9%) |
| Neonatal or perinatal ICU admission | 71 (16.0%) | 8 (20.5%) | 5 (55.6%) | 3 (42.9%) |
| Major congenital malformation | 40 (9.0%) | 4 (10.3%) | 1 (11.1%) | 1 (14.3%) |

Treatment categories are mutually exclusive and each pregnancy is counted once. Surgery refers to malignant tumor surgery recorded during pregnancy, which includes cervical procedures (K867 series); anticancer agent refers to at least one anticancer agent prescribed between cancer diagnosis and delivery. Radiation therapy during pregnancy was not recorded in any pregnancy. Primary cancer site is shown for the pregnancy as a whole, so pregnancies with more than one primary site are counted once and assigned to Other; counts therefore differ from Table 1, in which each primary site is counted separately. Hematologic malignancies comprise adult T-cell leukemia/lymphoma, non-Hodgkin lymphoma, Hodgkin lymphoma, acute and chronic leukemias, multiple myeloma and plasma cell neoplasms, and other hematopoietic tumors. Percentages were calculated within each category. Counts in the anticancer agent only and both categories are based on nine and seven pregnancies, respectively, and the corresponding percentages are unstable; they are shown for completeness and should not be interpreted as evidence of differences between categories. Formal statistical testing was not performed.
